# Long-term disability outcomes in Hansen’s disease: A 20-year comparative study of Lepromatous vs. Tuberculoid Leprosy

**DOI:** 10.1371/journal.pntd.0014385

**Published:** 2026-06-26

**Authors:** Elaine J. Ma, Brandon L. Adler, April W. Armstrong, Maria T. Ochoa

**Affiliations:** 1 University of Southern California Keck School of Medicine, Los Angeles, California, United States of America; 2 Department of Dermatology, University of Southern California Keck School of Medicine, Los Angeles, California, United States of America; 3 Division of Dermatology, Department of Medicine, David Geffen School of Medicine at the University of California, Los Angeles, California, United States of America; University of Bremen: Universitat Bremen, GERMANY

## Abstract

**Background:**

Leprosy remains an important cause of disability, even in non-endemic settings. Risk of neuropathy and functional impairment differs across the disease spectrum, yet contemporary U.S. data are limited.

**Objective:**

To compare disability-related outcomes among patients with lepromatous versus tuberculoid leprosy in a large U.S. electronic health record (EHR) network.

**Methods:**

Using the TriNetX U.S. Collaborative Research Network (2005–2025), patients with lepromatous (ICD-10-CM A30.4-A30.5) and tuberculoid (A30.1-A30.2) leprosy were identified. Disability-related outcomes were defined using ICD-10-CM and CPT codes. Patients with prior disability were excluded to assess incident events. Cohorts were propensity score-matched (1:1) on demographics and comorbidities. Odds ratios (OR) and hazard ratios (HR) with 95% confidence intervals (CI) were calculated.

**Results:**

A total of 341 lepromatous and 129 tuberculoid patients were identified (median follow-up 1,138 vs 1,021 days). Lepromatous leprosy was associated with higher risk of incident disability-related outcomes (OR 1.89, 95% CI 1.16-3.07). Kaplan-Meier analysis demonstrated lower outcome-free survival in the lepromatous cohort (HR 1.76, 95% CI 1.15-2.71).

**Conclusions:**

In this U.S.-based cohort, lepromatous leprosy was associated with greater disability-related morbidity compared to tuberculoid leprosy. However, meaningful risk was observed across both subtypes, supporting structured longitudinal follow-up for all patients.

## Author summary

Leprosy is often thought of as a disease of the past or limited to certain regions, but it continues to affect patients in the United States and can lead to long-term disability, particularly through nerve damage. Not all forms of leprosy behave the same way, and understanding these differences is important for improving patient care. In this study, we used a large U.S. electronic health record database to compare disability-related outcomes between two major forms of leprosy: lepromatous and tuberculoid. We found that patients with lepromatous leprosy had a higher risk of developing new disability-related complications over time compared to those with tuberculoid disease. However, patients in both groups experienced meaningful rates of disability, highlighting that all individuals with leprosy remain at risk. These findings suggest that while certain forms of leprosy may carry greater risk, careful and ongoing monitoring is important for every patient, regardless of subtype. Early recognition and management of complications may help reduce long-term disability and improve quality of life.

## Introduction

Leprosy is a debilitating, chronic infection of the skin and peripheral nerves that is associated with risk of disability. Although the number of cases has declined since the establishment of multidrug therapy, leprosy remains an important cause of disability, with approximately 175 cases reported annually in the US [1]. Leprosy can cause irreversible nerve damage, leading to loss of protective sensation, injuries, chronic ulcers, osteomyelitis, and need for amputation [2]. Risk of neuropathy and disability differs across the spectrum of leprosy from tuberculoid to lepromatous disease. Tuberculoid leprosy, typically paucibacillary and localized, contrasts with lepromatous leprosy, which is multibacillary, disseminated, and associated with a greater risk of nerve damage and disability [3]. Much of the literature on leprosy-associated disability comes from endemic regions, with few studies characterizing long-term disability outcomes in contemporary cohorts using real-world data. In this study, we used a large U.S.-based electronic health record (EHR) database to assess and compare disability-related outcomes among patients with lepromatous versus tuberculoid leprosy over a twenty-year observation period. We hypothesized that patients with lepromatous leprosy would have a higher risk of subsequent disability compared to those with tuberculoid leprosy.

## Methods

Using the TriNetX U.S. Collaborative Research Network (2005–2025), a national real-time EHR database, we identified patients with lepromatous and tuberculoid leprosy based on ICD-10-CM codes A30.4-A30.5 and A30.1-A30.2, respectively. We then evaluated disability-related outcomes using a set of International Classification of Diseases, Tenth Revision, Clinical Modification (ICD-10-CM) codes and Current Procedural Terminology (CPT codes) as proxies for functional impairment (Table 1). The index event was defined as the date of the patient’s initial leprosy diagnosis, marking their entry into the analysis. “Initial diagnosis” was operationalized in TriNetX as the first recorded occurrence of the relevant ICD-10-CM code within the network during the study period. Patients with any documented disability-related outcome included in the composite definition prior to the index date were excluded, regardless of attribution, to ensure assessment of incident events during follow-up. Lepromatous and tuberculoid patients were propensity score-matched on age, sex, race, ethnicity, and comorbidities including diabetes mellitus and tobacco use. Propensity score matching was performed using the TriNetX built-in algorithm, which applies 1:1 nearest-neighbor matching on the logit of the propensity score with a caliper of 0.1 pooled standard deviations and without replacement. Covariate balance was assessed using standardized mean differences (SMDs) before and after matching, with post-match SMDs < 0.1 considered indicative of adequate balance. Statistical analyses conducted within the TriNetX-analytics suite included odds ratios (OR) and hazard ratios (HR) with 95% confidence intervals (CI). Odds ratios (ORs) were calculated to estimate the association between exposure and incident disability within the defined follow-up period of twenty years after the index date. Analyses were conducted in the propensity score-matched cohorts, accounting for the matched design as implemented within the TriNetX platform. Time-to-event analyses were performed using Kaplan-Meier survival estimates within TriNetX. Hazard ratios (HRs) were generated by the platform using a Cox proportional hazards model applied to the matched cohorts. Patients were followed from the index date until the occurrence of the outcome or censoring at their last recorded encounter within the network. Right-censoring reflected loss to follow-up or end of available data. Proportional hazards assumptions were evaluated within the constraints of the TriNetX analytic framework.

**Table 1:** Disability-related outcomes defined by CPT and ICD-10-CM codes.

Disability-related outcome	CPT code
Amputation procedures of the foot and toes	1005524
Amputation procedures of the hands and fingers	1004834
Surgical interventions on the extracranial, peripheral, and autonomic nerves	1009526
**Disability-related outcome**	**ICD-10-CM code**
Polyneuropathies and other peripheral nervous system disorders	G60-G65
Blindness and low vision	H54
Non-pressure chronic ulcer of lower limb	L97
Contracture of joint (claw hand, foot drop)	M21.6X
Acquired deformity of nose (saddle nose deformity)	M95
Difficulty in walking due to disability	R26.2
Disability due to neurological disorder	R29.818
Problems related to employment due to disability	Z56
Acquired absence of limb	Z89.0-Z89.6
Dependence on wheelchair	Z99.3

## Results

**Fig 1:**
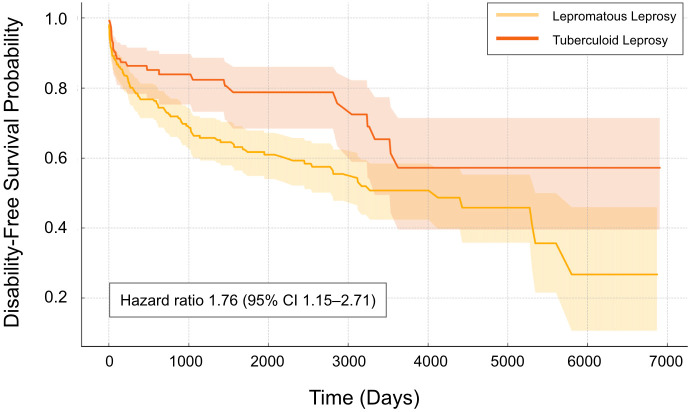
Kaplan-Meier survival analysis for disability-free survival in lepromatous and tuberculoid leprosy patient cohorts over twenty years with 95% confidence intervals.

A total of 341 individuals with lepromatous leprosy and 129 with tuberculoid leprosy were identified. The median follow-up duration was 1,138 days for the lepromatous leprosy cohort and 1,021 days for the tuberculoid leprosy cohort. Lepromatous leprosy patients had a nearly 90% higher risk of experiencing a disability-related outcome compared to tuberculoid leprosy patients [OR 1.89, 95% CI (1.16-3.07)]. Similarly, Kaplan Meier survival analysis demonstrated a difference in outcome-free survival between groups: 26.7% of lepromatous leprosy patients remained free from disability-related outcomes over a twenty year observation period, compared to 57.2% of tuberculoid leprosy patients (Fig 1). The hazard ratio for time to disability-related outcome was also significantly higher in the lepromatous cohort [HR 1.76, 95% CI (1.15–2.71)].

## Discussion

Over a twenty-year observation period, patients with lepromatous leprosy had a lower likelihood of remaining free from disability-related outcomes compared to tuberculoid leprosy patients. While this distinction exists, it is equally critical to recognize that all individuals affected by leprosy face a meaningful risk of disability over time. Limitations of our study include the long latency period of leprosy and frequent delayed diagnosis in non-endemic regions, which could contribute to underestimation of time to disability events [4,5]. Reliance on ICD-10-CM and CPT codes for the identification of both diagnoses and outcomes enables population-level analysis, but it may introduce misclassification bias due to coding variability. To prioritize sensitivity in detecting incident disability events, we included a broad set of codes informed by known complications of leprosy and patterns of functional decline observed in clinical practice. While some components may also be influenced by healthcare utilization or documentation practices, this approach is consistent with prior EHR-based outcomes research and allows for more comprehensive capture of disability-related morbidity. Importantly, all outcomes were assessed as incident events, with patients with documented disability prior to index excluded, reducing the likelihood that findings reflect pre-existing impairment. Nonetheless, we acknowledge that use of administrative proxy measures may introduce misclassification and should be interpreted in the context of inherent limitations of real-world data. Additionally, unmeasured confounders such as disease severity, socioeconomic status, and treatment adherence were not fully accounted for in our analysis. As with all retrospective EHR-based survival analyses, our findings are subject to inherent limitations, including variable and potentially differential follow-up across groups, which may influence time-to-event estimates despite the use of censoring methods. Further studies should incorporate clinical severity measures, longitudinal functional assessments, and patient-reported quality-of-life data to better characterize disability progression in leprosy.

In non-endemic settings, these findings underscore the importance of structured longitudinal follow-up for all patients with leprosy, as both lepromatous and tuberculoid subtypes carry risk of subsequent disability-related complications. Although patients with lepromatous disease demonstrated a higher relative burden, individuals with tuberculoid disease also experienced meaningful rates of disability-related outcomes, supporting the need for ongoing surveillance across the disease spectrum. Clinicians should maintain vigilance for early signs of neuropathy, ulceration, vision-related complications, and other functional impairments, with timely referral to multidisciplinary services, including dermatology, neurology, wound care, and rehabilitation. Ongoing disability risk observed over extended follow-up may reflect delayed neuropathic progression, chronic inflammatory reactions, or cumulative nerve damage that can persist or evolve even after initial diagnosis and completion of antimicrobial therapy. These findings reinforce the importance of sustained follow-up beyond completion of antimicrobial therapy, even in low-incidence, non-endemic settings where familiarity with long-term sequelae may be limited.

**Ethical Approval:** The study was performed in full accordance with the Helsinki Declaration of 1975, as revised in 1983. The analysis was conducted using publicly available data, and therefore did not require institutional review board approval.

**Ethics Statement:** The study used de-identified and aggregate data from electronic medical records that did not require Institutional Review Board approval or written informed consent.
