# Supplementary material for: Long-term disability outcomes in Hansen’s disease: A 20-year comparative study of Lepromatous vs. Tuberculoid Leprosy
Source: PLoS Negl Trop Dis. 2026 Jun 26;20(6):e0014385. doi: 10.1371/journal.pntd.0014385 (PMC13308791; doi:10.1371/journal.pntd.0014385)
Supplement: Attachment — Submitted filename: Response to Reviewers (3.5.26).pdf [file pntd.0014385.s001.pdf]

Reviewer #1: The objectives are clear.

Reviewer #2: Objectives nit clearly stated but appear to be the comparison of disability risk between tuberculoid and lepromatous leprosy patients.

Reviewer #3: The study objective is clear, but the manuscript would benefit from an explicit, testable hypothesis (e.g., that lepromatous disease is associated with higher subsequent disability risk than tuberculoid disease).

The cohort definitions need more detail. Please report the exact ICD-10-CM codes used to identify lepromatous and tuberculoid leprosy and define precisely how "initial diagnosis" is operationalized in TriNetX (e.g., first-ever record in the network versus first within a fixed lookback period). Please also describe how patients were handled if their diagnosis subtype changed over time (e.g., recoding from tuberculoid to lepromatous or vice versa), and whether any exclusion rules were applied.

Thank you for your insightful comment. We have revised the manuscript to include an explicit, testable hypothesis: we hypothesized that patients with lepromatous leprosy would have a higher risk of subsequent disability compared to those with tuberculoid leprosy (line 113-114).

We have also expanded the Methods section to clarify cohort definitions. "Initial diagnosis" was operationalized in TriNetX as the first recorded occurrence of the relevant ICD-10-CM code within the network during the study period (line 121-123). In the TriNetX research platform, diagnosis subtype changes over time cannot be reliably ascertained, as the dataset does not provide longitudinal adjudication of re-coding or a structured indicator of subtype transition. Therefore, we were unable to determine whether patients were later reclassified from tuberculoid to lepromatous disease (or vice versa). Patients were categorized based on their first recorded ICD-10-CM code subtype at the time of cohort entry, as defined in the Methods. To assess incident disability risk, patients with any record of the outcome of interest prior to cohort entry (index date) were excluded (lines 124-126).

The main outcome definition is a major concern. "Disability-related outcomes" are defined as a composite of ICD and procedure proxies (Table I), but several listed elements appear non-specific and may reflect social/administrative circumstances rather than clinical disability. The manuscript should (i) justify clinical relevance for each included code, (ii) clarify whether outcomes were treated as incident versus prevalent, (iii) specify any confirmation rules (e.g., ≥2 occurrences, separation in time), and (iv) report which codes contribute most strongly to the composite. A more clinically anchored primary composite (e.g., neuropathy/neuritis, chronic ulceration, amputations, contractures/deformities, vision-threatening complications) with a broader secondary composite would substantially improve interpretability.

Thank you for this thoughtful critique. The composite outcome was intentionally designed to capture the multidimensional nature of disability as recorded in real-world data, where functional impairment is often reflected through a combination of diagnostic and procedural codes rather than a single clinically uniform endpoint. We have clarified in the Methods that outcomes were assessed as incident events, with patients with documented disability prior to index excluded.

Given the constraints of administrative coding within TriNetX, we did not apply additional confirmation rules (e.g., ≥2 occurrences), as this may reduce sensitivity for clinically meaningful events. We have added text to better justify the clinical relevance of the included codes and to acknowledge the limitations of using proxy measures in EHR-based research. We believe this approach preserves sensitivity while

reflecting real-world disability documentation patterns, and we have expanded the Discussion to address interpretability considerations (line 162-171).

The propensity score matching approach is insufficiently described. Please specify the matching ratio, algorithm (e.g., nearest neighbor), caliper, use of replacement, and the index date used for time zero. Balance diagnostics are essential: provide standardized mean differences (SMDs) for each covariate pre- and post-match (table or plot). If follow-up duration differs meaningfully between groups, consider approaches that address informative censoring or differential healthcare utilization.

Thank you for this important comment. We have expanded the Methods (lines 128-132) to clarify that propensity score matching was performed using the TriNetX built-in algorithm, which applies 1:1 nearest-neighbor matching on the logit of the propensity score with a caliper of 0.1 pooled standard deviations and without replacement. The index date (time zero) was defined as the date of the first qualifying diagnosis (cohort entry). Standardized mean differences (SMDs) for each covariate before and after matching, to assess covariate balance, met conventional thresholds indicative of adequate balance (SMD < 0.1). In TriNetX Kaplan-Meier survival analyses, patients are followed from the defined index date (time zero) until the earliest of: (1) occurrence of the outcome of interest, or (2) the last recorded encounter in the network. Patients who do not experience the outcome are right-censored at their last known activity in the EHR. TriNetX does not apply additional adjustment for informative censoring; censoring is based solely on loss to follow-up or end of available data within the network. We clarified this in our methods section (lines 138-143).

The statistical methods require clearer reporting. The manuscript reports both odds ratios and hazard ratios, but does not clearly define the time window associated with the OR outcome or whether matched-pair structure was accounted for in the OR estimation. For Cox models, please specify censoring rules, how loss to follow-up was handled, and whether proportional hazards assumptions were evaluated.

Thank you very much for this comment. We have clarified the statistical methods in the revised manuscript. Specifically, we now define the time window associated with the odds ratio analysis and clarify how the matched design was accounted for in its estimation (line 134-143). For the Cox proportional hazards models, we explicitly describe the censoring rules, handling of loss to follow-up, and definition of time zero. We also note that proportional hazards assumptions were assessed within the constraints of the TriNetX platform.

Results

Reviewer #1: The results are as expected.

Reviewer #2: Results are clearly presented but the analysis is rather basic

Reviewer #3: The manuscript emphasizes “20-year outcome-free survival,” yet the reported median follow-up is approximately 3 years (1,138 and 1,021 days). This raises concern that the Kaplan–Meier estimates at longer time horizons may be driven by a very small number of individuals remaining at risk and thus be unstable. To support any long-term claims, please provide numbers at risk at prespecified time points (e.g., 1, 5, 10, and 20 years), and ideally include censor marks on the survival curves. If the number at risk becomes very small at later time points, the manuscript should either, avoid emphasizing a

20-year horizon, or clearly qualify the estimates as imprecise due to censoring and sparse late follow-up.

In addition, please report the post-matching cohort sizes and outcome event counts in each group, and consider presenting incidence rates (events per person-years) alongside HR/OR estimates. These additions would make the magnitude and clinical relevance of the association more transparent.

Thank you for your insightful suggestions. We clarify that the study does not reflect 20 years of median follow-up, but rather a 20-year observation window within the TriNetX network during which eligible patients could enter the cohort (line 113,150, and 153). The median follow-up duration was 1,138 days for the lepromatous cohort and 1,021 days for the tuberculoid cohort. Kaplan-Meier methodology accounts for variable follow-up and right-censoring, though we acknowledge that estimates at later time points reflect progressively fewer individuals at risk and should be interpreted accordingly. We believe that the hazard ratios and odds ratios provide the most clinically interpretable measures of association within the matched design; however, we clarified in the discussion the inherent limitations of retrospective EHR-based survival analyses, particularly with respect to differential follow-up (lines 173-175)

Conclusions

Reviewer #1: There don't seem to be any conclusions, except to say that it would be better to use more comprehensive measures of disability, including functional assessments, in future studies.

Reviewer #2: The conclusions do match the data presented

Reviewer #3: The conclusion that lepromatous leprosy is associated with a higher burden of subsequent disability-related outcomes than tuberculoid leprosy is directionally plausible and consistent with clinical expectations. However, the strength and long-term framing of the conclusions are not fully supported given the breadth and limited specificity of the composite outcome, and the apparent mismatch between median follow-up and the emphasized 20-year survival estimates. The authors should temper long-term inferences unless supported by adequate numbers at risk over time, and should clearly describe the outcome as a disability-related proxy composite.

Public health and clinical relevance are appropriate for a non-endemic setting; the discussion would benefit from more concrete implications for follow-up care (e.g., monitoring for neuropathy/ulceration, referral pathways for disability prevention) while acknowledging limited generalizability to endemic program settings.

Thank you for this thoughtful feedback. We have revised the manuscript's Discussion to more precisely describe the primary endpoint as a disability-related proxy composite and to moderate long-term framing of the findings. We clarify that the 20-year period reflects the observation window rather than median follow-up duration and emphasize appropriate caution in interpreting later time-point estimates.

We have also refined the Discussion to focus on practical clinical implications in non-endemic settings, including the importance of structured follow-up for neuropathy and ulceration monitoring and early referral for disability prevention, while acknowledging limitations in generalizability to endemic program contexts (line 178-191).

Editorial and Data Presentation Modifications

Use this section for editorial suggestions as well as relatively minor modifications of existing data that would enhance clarity. If the only modifications needed are minor and/or editorial, you may wish to recommend “Minor Revision” or “Accept”.

Reviewer #1: I was once told that a paper I had written was 'too succinct'. This paper challenges the record for being the most succinct I have ever seen.

There are several aspects of the paper that could be expanded with more complete explanations:
1. The list of abbreviations could include US, TriNetX, ICD-10-CM, CPT, and CI. Since there is so much space, why not include more of an explanation for these terms?
2. Please explain in more detail the sentence "Patients with prior disability outcomes were excluded." Does this refer to non-leprosy-related disability or old leprosy-related disabilities, or both?
3. The median follow-up period was just over 1000 days for both groups, so large numbers in both groups must have either died or been lost to follow-up. Thus the figures of 26.7% and 57.2% disability-free survival at 20 years are meaningless.

Thank you for these constructive comments.

1. We have expanded the list of abbreviations and clarified definitions at first mention to improve clarity for readers less familiar with administrative datasets.

2. We have clarified that exclusion of “prior disability outcomes” refers to any documented disability-related outcome included in our composite definition occurring before the index date, regardless of whether it was directly attributed to leprosy. This approach was used to ensure capture of incident events during follow-up (line 124-126)

3. We agree that the median follow-up duration (1,138 and 1,021 days, respectively) differs from the 20-year observation window. We have clarified that the 20-year timeframe reflects the maximum available observation period within the network rather than median follow-up, and we have moderated language surrounding long-term estimates to emphasize appropriate interpretation given progressive censoring over time.

Reviewer #2: (No Response)

Reviewer #3: Please ensure the manuscript is internally consistent regarding article type (the submission appears to mix “Research Article” language with a “Research Letter/Brief Report” format and strict word limits). Consistency with the journal’s category requirements is needed.

Thank you for this observation. We have reviewed the manuscript and revised it to ensure consistency with the journal’s designated article category, including alignment of formatting, structure, and language with the appropriate submission type.

Summary and General Comments

Use this section to provide overall comments, discuss strengths/weaknesses of the study, novelty, significance, general execution and scholarship. You may also include additional comments for the author, including concerns about dual publication, research ethics, or publication ethics. If requesting major revision, please articulate the new experiments that are needed.

Reviewer #1: (No Response)

Reviewer #2: Long-Term Disability Outcomes in Hansen's Disease: A 20-Year Comparative Study of Lepromatous vs. Tuberculoid Leprosy
The authors performed a retrospective cohort study in which they compared 431 lepromatous leprosy patients to 129 tuberculoid leprosy patients, all diagnosed in the US over the period of 2005-2025 and available from a database. Their main finding is that lepromatous leprosy was associated with a higher risk of disability-related outcomes (OR 1.89, 95% CI 1.16-3.07). They conclude that lepromatous leprosy was associated with a substantially higher risk of disability-related outcomes compared with tuberculoid leprosy.

Major comments:
These findings are certainly correct but there's nothing in them that was not already known.
The authors distinguish between tuberculoid and lepromatous leprosy but if they wish to use the Ridley-Jopling classification, they should consider all five categories. How were borderline-tuberculoid, mid-borderline and borderline-lepromatous patients classified? For operational reasons it would be more meaningful to distinguish between multi- and paucibacillary leprosy, where probably the cutoff will be somewhere within the borderline-tuberculoid group.

The Kaplan-Meier graph is interesting because it shows how even after 10 years of follow-up new disabilities occur. It would be interesting to know more about the reasons behind this.

Thank you for your comprehensive insights. We agree that the observed directional differences are clinically consistent with existing understanding; however, our aim was to quantify this association in a contemporary, non-endemic U.S. population using real-world data, where longitudinal outcome data remain limited. We have clarified this contribution in the Introduction and Discussion.

Regarding classification, our cohorts were defined strictly using ICD-10-CM codes corresponding to tuberculoid and lepromatous leprosy. The TriNetX dataset does not reliably capture the full Ridley-Jopling spectrum (e.g., borderline-tuberculoid, mid-borderline, borderline-lepromatous) in a granular or consistently coded manner. Therefore, we were unable to stratify across all five Ridley-Jopling categories or operationally reclassify cases as multibacillary versus paucibacillary.

We agree that the persistence of incident disability over extended follow-up is clinically meaningful. As TriNetX does not provide detailed clinical adjudication of underlying mechanisms, we cannot directly determine causation. However, we have expanded the Discussion to note that delayed neuropathic progression, chronic inflammatory reactions, and cumulative nerve damage may contribute to ongoing disability risk even years after initial diagnosis and treatment (lines 186-188).

Reviewer #3: This is a timely and potentially publishable brief report addressing an important gap in knowledge about disability-related outcomes among leprosy patients in a non-endemic setting. Use of a large multi-institution EHR network and an attempt to apply matching and survival analysis are strengths.

However, I recommend Major Revision because key methodological and reporting issues currently limit interpretability and reproducibility.

Thank you for your valuable insights and constructive evaluation. We appreciate your recognition of the study's relevance and methodological strengths. We have carefully addressed the key methodological and reporting concerns raised and have revised the manuscript to improve clarity, transparency, and

reproducibility. We hope the revisions adequately resolve these issues and strengthen the overall interpretability of the work. Thank you again for your time and consideration.
