# Supplementary material for: Long-term disability outcomes in Hansen’s disease: A 20-year comparative study of Lepromatous vs. Tuberculoid Leprosy
Source: PLoS Negl Trop Dis. 2026 Jun 26;20(6):e0014385. doi: 10.1371/journal.pntd.0014385 (PMC13308791; doi:10.1371/journal.pntd.0014385)
Supplement: Peer review history

# Peer review: Decision Letter 0

**DOI:** 10.1371/journal.pntd.0014385.r001

**Authors:** Joseph Vinetz, Anil Fastenau

27 Feb 2026

PNTD-D-26-00253

Long-Term Disability Outcomes in Hansen’s Disease: A 20-Year Comparative Study of Lepromatous vs. Tuberculoid Leprosy

PLOS Neglected Tropical Diseases

Dear Dr. Ma,

Thank you for submitting your manuscript to PLOS Neglected Tropical Diseases. After careful consideration, we feel that it has merit but does not fully meet PLOS Neglected Tropical Diseases’s publication criteria as it currently stands. Therefore, we invite you to submit a revised version of the manuscript that addresses the points raised during the review process.

Please submit your revised manuscript within by Apr 28 2026 11:59PM. If you will need more time than this to complete your revisions, please reply to this message or contact the journal office at plosntds@plos.org. When you’re ready to submit your revision, log on to https://www.editorialmanager.com/pntd/ and select the ‘Submissions Needing Revision’ folder to locate your manuscript file.

Please include the following items when submitting your revised manuscript:

* A letter that responds to each point raised by the editor and reviewer(s). You should upload this letter as a separate file labeled ‘Response to Reviewers’. This file does not need to include responses to any formatting updates and technical items listed in the ‘Journal Requirements’ section below.

* A marked-up copy of your manuscript that highlights changes made to the original version. You should upload this as a separate file labeled ‘Revised Manuscript with Track Changes’.

* An unmarked version of your revised paper without tracked changes. You should upload this as a separate file labeled ‘Manuscript’.

If you would like to make changes to your financial disclosure, competing interests statement, or data availability statement, please make these updates within the submission form at the time of resubmission. Guidelines for resubmitting your figure files are available below the reviewer comments at the end of this letter.

We look forward to receiving your revised manuscript.

Kind regards,

Anil Fastenau, M.D., M.Sc.

Guest Editor

PLOS Neglected Tropical Diseases

Joseph Vinetz

Section Editor

PLOS Neglected Tropical Diseases

Shaden Kamhawi

co-Editor-in-Chief

PLOS Neglected Tropical Diseases

orcid.org/0000-0003-4304-636XX

Paul Brindley

co-Editor-in-Chief

PLOS Neglected Tropical Diseases

orcid.org/0000-0003-1765-0002

**Journal Requirements:**

1) Please ensure that the CRediT author contributions listed for every co-author are completed accurately and in full.

At this stage, the following Authors/Authors require contributions: Elaine J. Ma, Brandon L. Adler, April W. Armstrong, and Maria T. Ochoa. Please ensure that the full contributions of each author are acknowledged in the "Add/Edit/Remove Authors" section of our submission form.

The list of CRediT author contributions may be found here: https://journals.plos.org/plosntds/s/authorship#loc-author-contributions

2) We ask that a manuscript source file is provided at Revision. Please upload your manuscript file as a .doc, .docx, .rtf or .tex. If you are providing a .tex file, please upload it under the item type u2018LaTeX Source Fileu2019 and leave your .pdf version as the item type u2018Manuscriptu2019.

3) Please provide an Author Summary. This should appear in your manuscript between the Abstract (if applicable) and the Introduction, and should be 150-200 words long. The aim should be to make your findings accessible to a wide audience that includes both scientists and non-scientists. Sample summaries can be found on our website under Submission Guidelines:

https://journals.plos.org/plosntds/s/submission-guidelines#loc-parts-of-a-submission

4) Please upload all main figures as separate Figure files in .tif or .eps format. For more information about how to convert and format your figure files please see our guidelines:

https://journals.plos.org/plosntds/s/figures

5) We note that your Data Availability Statement is currently as follows: "The data that support the findings of this study are available from TriNetX. Restrictions apply to the availability of these data, which were used under license for this study.". Please confirm at this time whether or not your submission contains all raw data required to replicate the results of your study. Authors must share the “minimal data set” for their submission. PLOS defines the minimal data set to consist of the data required to replicate all study findings reported in the article, as well as related metadata and methods (https://journals.plos.org/plosone/s/data-availability#loc-minimal-data-set-definition).

For example, authors should submit the following data:

1) The values behind the means, standard deviations and other measures reported;

2) The values used to build graphs;

3) The points extracted from images for analysis..

Authors do not need to submit their entire data set if only a portion of the data was used in the reported study.

If your submission does not contain these data, please either upload them as Supporting Information files or deposit them to a stable, public repository and provide us with the relevant URLs, DOIs, or accession numbers. For a list of recommended repositories, please see https://journals.plos.org/plosone/s/recommended-repositories.

If there are ethical or legal restrictions on sharing a de-identified data set, please explain them in detail (e.g., data contain potentially sensitive information, data are owned by a third-party organization, etc.) and who has imposed them (e.g., an ethics committee). Please also provide contact information for a data access committee, ethics committee, or other institutional body to which data requests may be sent. If data are owned by a third party, please indicate how others may request data access.

**Reviewers’ Comments:**

Reviewer’s Responses to Questions

**Key Review Criteria Required for Acceptance?**

As you describe the new analyses required for acceptance, please consider the following:

**Methods**

-Are the objectives of the study clearly articulated with a clear testable hypothesis stated?

-Is the study design appropriate to address the stated objectives?

-Is the population clearly described and appropriate for the hypothesis being tested?

-Is the sample size sufficient to ensure adequate power to address the hypothesis being tested?

-Were correct statistical analysis used to support conclusions?

-Are there concerns about ethical or regulatory requirements being met?

Reviewer #1: The objectives are clear.

Reviewer #2: Objectives nit clearly stated but appear to be the comparison of disability risk between tuberculoid and lepromatous leprosy patients.

Reviewer #3: The study objective is clear, but the manuscript would benefit from an explicit, testable hypothesis (e.g., that lepromatous disease is associated with higher subsequent disability risk than tuberculoid disease).

The cohort definitions need more detail. Please report the exact ICD-10-CM codes used to identify lepromatous and tuberculoid leprosy and define precisely how “initial diagnosis” is operationalized in TriNetX (e.g., first-ever record in the network versus first within a fixed lookback period). Please also describe how patients were handled if their diagnosis subtype changed over time (e.g., recoding from tuberculoid to lepromatous or vice versa), and whether any exclusion rules were applied.

The main outcome definition is a major concern. “Disability-related outcomes” are defined as a composite of ICD and procedure proxies (Table I), but several listed elements appear non-specific and may reflect social/administrative circumstances rather than clinical disability. The manuscript should (i) justify clinical relevance for each included code, (ii) clarify whether outcomes were treated as incident versus prevalent, (iii) specify any confirmation rules (e.g., ≥2 occurrences, separation in time), and (iv) report which codes contribute most strongly to the composite. A more clinically anchored primary composite (e.g., neuropathy/neuritis, chronic ulceration, amputations, contractures/deformities, vision-threatening complications) with a broader secondary composite would substantially improve interpretability.

The propensity score matching approach is insufficiently described. Please specify the matching ratio, algorithm (e.g., nearest neighbor), caliper, use of replacement, and the index date used for time zero. Balance diagnostics are essential: provide standardized mean differences (SMDs) for each covariate pre- and post-match (table or plot). If follow-up duration differs meaningfully between groups, consider approaches that address informative censoring or differential healthcare utilization.

The statistical methods require clearer reporting. The manuscript reports both odds ratios and hazard ratios, but does not clearly define the time window associated with the OR outcome or whether matched-pair structure was accounted for in the OR estimation. For Cox models, please specify censoring rules, how loss to follow-up was handled, and whether proportional hazards assumptions were evaluated.

**********

**Results**

-Does the analysis presented match the analysis plan?

-Are the results clearly and completely presented?

-Are the figures (Tables, Images) of sufficient quality for clarity?

Reviewer #1: The results are as expected.

Reviewer #2: Results are clearly presented but the analysis is rather basic

Reviewer #3: The manuscript emphasizes “20-year outcome-free survival,” yet the reported median follow-up is approximately 3 years (1,138 and 1,021 days). This raises concern that the Kaplan–Meier estimates at longer time horizons may be driven by a very small number of individuals remaining at risk and thus be unstable. To support any long-term claims, please provide numbers at risk at prespecified time points (e.g., 1, 5, 10, and 20 years), and ideally include censor marks on the survival curves. If the number at risk becomes very small at later time points, the manuscript should either, avoid emphasizing a 20-year horizon, or clearly qualify the estimates as imprecise due to censoring and sparse late follow-up.

In addition, please report the post-matching cohort sizes and outcome event counts in each group, and consider presenting incidence rates (events per person-years) alongside HR/OR estimates. These additions would make the magnitude and clinical relevance of the association more transparent.

**********

**Conclusions**

-Are the conclusions supported by the data presented?

-Are the limitations of analysis clearly described?

-Do the authors discuss how these data can be helpful to advance our understanding of the topic under study?

-Is public health relevance addressed?

Reviewer #1: There don’t seem to be any conclusions, except to say that it would be better to use more comprehensive measures of disability, including functional assessments, in future studies.

Reviewer #2: The conclusions do match the data presented

Reviewer #3: The conclusion that lepromatous leprosy is associated with a higher burden of subsequent disability-related outcomes than tuberculoid leprosy is directionally plausible and consistent with clinical expectations. However, the strength and long-term framing of the conclusions are not fully supported given the breadth and limited specificity of the composite outcome, and the apparent mismatch between median follow-up and the emphasized 20-year survival estimates. The authors should temper long-term inferences unless supported by adequate numbers at risk over time, and should clearly describe the outcome as a disability-related proxy composite.

Public health and clinical relevance are appropriate for a non-endemic setting; the discussion would benefit from more concrete implications for follow-up care (e.g., monitoring for neuropathy/ulceration, referral pathways for disability prevention) while acknowledging limited generalizability to endemic program settings.

**********

**Editorial and Data Presentation Modifications?**

Use this section for editorial suggestions as well as relatively minor modifications of existing data that would enhance clarity. If the only modifications needed are minor and/or editorial, you may wish to recommend “Minor Revision” or “Accept”.

Reviewer #1: I was once told that a paper I had written was ‘too succinct’. This paper challenges the record for being the most succinct I have ever seen.

There are several aspects of the paper that could be expanded with more complete explanations:

1. The list of abbreviations could include US, TriNetX, ICD-10-CM, CPT, and CI. Since there is so much space, why not include more of an explanation for these terms?

2. Please explain in more detail the sentence "Patients with prior disability outcomes were excluded." Does this refer to non-leprosy-related disability or old leprosy-related disabilities, or both?

3. The median follow-up period was just over 1000 days for both groups, so large numbers in both groups must have either died or been lost to follow-up. Thus the figures of 26.7% and 57.2% disability-free survival at 20 years are meaningless.

Reviewer #2: (No Response)

Reviewer #3: Please ensure the manuscript is internally consistent regarding article type (the submission appears to mix “Research Article” language with a “Research Letter/Brief Report” format and strict word limits). Consistency with the journal’s category requirements is needed.

**********

**Summary and General Comments**

Use this section to provide overall comments, discuss strengths/weaknesses of the study, novelty, significance, general execution and scholarship. You may also include additional comments for the author, including concerns about dual publication, research ethics, or publication ethics. If requesting major revision, please articulate the new experiments that are needed.

Reviewer #1: (No Response)

Reviewer #2: Long-Term Disability Outcomes in Hansen’s Disease: A 20-Year Comparative Study of Lepromatous vs. Tuberculoid Leprosy

The authors performed a retrospective cohort study in which they compared 431 lepromatous leprosy patients to 129 tuberculoid leprosy patients, all diagnosed in the US over the period of 2005-2025 and available from a database. Their main finding is that lepromatous leprosy was associated with a higher risk of disability-related outcomes (OR 1.89, 95% CI 1.16-3.07). They conclude that lepromatous leprosy was associated with a substantially higher risk of disability-related outcomes compared with tuberculoid leprosy.

Major comments:

These findings are certainly correct but there’s nothing in them that was not already known.

The authors distinguish between tuberculoid and lepromatous leprosy but if they wish to use the Ridley-Jopling classification, they should consider all five categories. How were borderline-tuberculoid, mid-borderline and borderline-lepromatous patients classified? For operational reasons it would be more meaningful to distinguish between multi- and paucibacillary leprosy, where probably the cutoff will be somewhere within the borderline-tuberculoid group.

The Kaplan-Meier graph is interesting because it shows how even after 10 years of follow-up new disabilities occur. It would be interesting to know more about the reasons behind this.

Reviewer #3: This is a timely and potentially publishable brief report addressing an important gap in knowledge about disability-related outcomes among leprosy patients in a non-endemic setting. Use of a large multi-institution EHR network and an attempt to apply matching and survival analysis are strengths.

However, I recommend Major Revision because key methodological and reporting issues currently limit interpretability and reproducibility.

**********

PLOS authors have the option to publish the peer review history of their article (what does this mean?). If published, this will include your full peer review and any attached files.

If you choose “no”, your identity will remain anonymous but your review may still be made public.

**Do you want your identity to be public for this peer review?**  For information about this choice, including consent withdrawal, please see our Privacy Policy.

Reviewer #1: No

Reviewer #2: No

Reviewer #3: No

[NOTE: If reviewer comments were submitted as an attachment file, they will be attached to this email and accessible via the submission site. Please log into your account, locate the manuscript record, and check for the action link "View Attachments". If this link does not appear, there are no attachment files.]

**Figure resubmission:**

While revising your submission, we strongly recommend that you use PLOS’s NAAS tool (https://ngplosjournals.pagemajik.ai/artanalysis) to test your figure files. NAAS can convert your figure files to the TIFF file type and meet basic requirements (such as print size, resolution), or provide you with a report on issues that do not meet our requirements and that NAAS cannot fix.

After uploading your figures to PLOS’s NAAS tool - https://ngplosjournals.pagemajik.ai/artanalysis, NAAS will process the files provided and display the results in the "Uploaded Files" section of the page as the processing is complete. If the uploaded figures meet our requirements (or NAAS is able to fix the files to meet our requirements), the figure will be marked as "fixed" above. If NAAS is unable to fix the files, a red "failed" label will appear above. When NAAS has confirmed that the figure files meet our requirements, please download the file via the download option, and include these NAAS processed figure files when submitting your revised manuscript.

**Reproducibility:**

To enhance the reproducibility of your results, we recommend that authors of applicable studies deposit laboratory protocols in protocols.io, where a protocol can be assigned its own identifier (DOI) such that it can be cited independently in the future. Additionally, PLOS ONE offers an option to publish peer-reviewed clinical study protocols. Read more information on sharing protocols at https://plos.org/protocols?utm_medium=editorial-email&utm_source=authorletters&utm_campaign=protocols

# Author comment: Author response to Decision Letter 1

**DOI:** 10.1371/journal.pntd.0014385.r002

2 Apr 2026

Attachment Submitted filename: Response to Reviewers (3.5.26).pdf

# Peer review: Decision Letter 1

**DOI:** 10.1371/journal.pntd.0014385.r003

**Authors:** Joseph Vinetz, Anil Fastenau, Joseph Vinetz, Anil Fastenau

15 May 2026

Dear Ms. Ma,

We are pleased to inform you that your manuscript ‘Long-Term Disability Outcomes in Hansen’s Disease: A 20-Year Comparative Study of Lepromatous vs. Tuberculoid Leprosy’ has been provisionally accepted for publication in PLOS Neglected Tropical Diseases.

Before your manuscript can be formally accepted you will need to complete some formatting changes, which you will receive in a follow up email. A member of our team will be in touch with a set of requests.

Please note that your manuscript will not be scheduled for publication until you have made the required changes, so a swift response is appreciated.

IMPORTANT: The editorial review process is now complete. PLOS will only permit corrections to spelling, formatting or significant scientific errors from this point onwards. Requests for major changes, or any which affect the scientific understanding of your work, will cause delays to the publication date of your manuscript.

Should you, your institution’s press office or the journal office choose to press release your paper, you will automatically be opted out of early publication. We ask that you notify us now if you or your institution is planning to press release the article. All press must be co-ordinated with PLOS.

Thank you again for supporting Open Access publishing; we are looking forward to publishing your work in PLOS Neglected Tropical Diseases.

Best regards,

Anil Fastenau, M.D., M.Sc.

Guest Editor

PLOS Neglected Tropical Diseases

Joseph Vinetz

Section Editor

PLOS Neglected Tropical Diseases

Shaden Kamhawi

co-Editor-in-Chief

PLOS Neglected Tropical Diseases

orcid.org/0000-0003-4304-636XX

Paul Brindley

co-Editor-in-Chief

PLOS Neglected Tropical Diseases

orcid.org/0000-0003-1765-0002

***********************************************************

p.p1 {margin: 0.0px 0.0px 0.0px 0.0px; line-height: 16.0px; font: 14.0px Arial; color: #323333; -webkit-text-stroke: #323333}span.s1 {font-kerning: none

Reviewer’s Responses to Questions

**Key Review Criteria Required for Acceptance?**

As you describe the new analyses required for acceptance, please consider the following:

**Methods**

-Are the objectives of the study clearly articulated with a clear testable hypothesis stated?

-Is the study design appropriate to address the stated objectives?

-Is the population clearly described and appropriate for the hypothesis being tested?

-Is the sample size sufficient to ensure adequate power to address the hypothesis being tested?

-Were correct statistical analysis used to support conclusions?

-Are there concerns about ethical or regulatory requirements being met?

Reviewer #3: (No Response)

Reviewer #4: The revised version clearly states the objective, definitions and methodology are clear.

No concerns about ethical/ regulatory requirements

Reviewer #5: There are lack of many information such as how the 341 LL and 129 t leprosy patients in a database most readers not familiar.

Reviewer #6: The objective of the study and the study design are appropriate. The authors have improved the description of cohort selection and analysis well and the methodology appears clear.

**********

**Results**

-Does the analysis presented match the analysis plan?

-Are the results clearly and completely presented?

-Are the figures (Tables, Images) of sufficient quality for clarity?

Reviewer #3: (No Response)

Reviewer #4: The results are appropriately presented .

Reviewer #5: The results were too short and far to draw the conclusions.

Reviewer #6: The figures and tables are clear and the results are clearly presented and consistent with study plan.

**********

**Conclusions**

-Are the conclusions supported by the data presented?

-Are the limitations of analysis clearly described?

-Do the authors discuss how these data can be helpful to advance our understanding of the topic under study?

-Is public health relevance addressed?

Reviewer #3: (No Response)

Reviewer #4: The conclusions, may be expected, but the short report is timely as discussion in the leprosy world about long term follow up to help with prevention of disability are picking up momentum.

The limitations are clearly discussed

Reviewer #5: The paper is not qualified as an article as well as a complete study.

Reviewer #6: Following the revision, there is appropriate discussion on limitations and the conclusions are consistent with known clinical patterns.

**********

**Editorial and Data Presentation Modifications?**

Use this section for editorial suggestions as well as relatively minor modifications of existing data that would enhance clarity. If the only modifications needed are minor and/or editorial, you may wish to recommend “Minor Revision” or “Accept”.

Reviewer #3: (No Response)

Reviewer #4: none

Reviewer #5: NA

Reviewer #6: (No Response)

**********

**Summary and General Comments**

Use this section to provide overall comments, discuss strengths/weaknesses of the study, novelty, significance, general execution and scholarship. You may also include additional comments for the author, including concerns about dual publication, research ethics, or publication ethics. If requesting major revision, please articulate the new experiments that are needed.

Reviewer #3: (No Response)

Reviewer #4: as above

Reviewer #5: The manuscript raised an important questions and the study design is good. However, the author didn’t review enough previous studies in the background. Secondly but more important, there are many aspects need to clarify in the results part especially for the patients characteristics. The author should expand the introduction and results part to be an article.

Reviewer #6: Overall, the feedback from previous reviewers has been well incorporated and addressed. There are no further comments.

**********

PLOS authors have the option to publish the peer review history of their article (what does this mean?). If published, this will include your full peer review and any attached files.

If you choose “no”, your identity will remain anonymous but your review may still be made public.

**Do you want your identity to be public for this peer review?**  For information about this choice, including consent withdrawal, please see our Privacy Policy.

Reviewer #3: No

Reviewer #4: No

Reviewer #5: No

Reviewer #6: No

# Editor report: Acceptance letter

**DOI:** 10.1371/journal.pntd.0014385.r004

**Authors:** Joseph Vinetz, Anil Fastenau, Joseph Vinetz, Anil Fastenau

Dear Ms. Ma,

We are delighted to inform you that your manuscript, "Long-Term Disability Outcomes in Hansen’s Disease: A 20-Year Comparative Study of Lepromatous vs. Tuberculoid Leprosy," has been formally accepted for publication in PLOS Neglected Tropical Diseases.

We have now passed your article onto the PLOS Production Department who will complete the rest of the publication process. All authors will receive a confirmation email upon publication.

The corresponding author will soon be receiving a typeset proof for review, to ensure errors have not been introduced during production. Please review the PDF proof of your manuscript carefully, as this is the last chance to correct any scientific or type-setting errors. Please note that major changes, or those which affect the scientific understanding of the work, will likely cause delays to the publication date of your manuscript. Note: Proofs for Front Matter articles (Editorial, Viewpoint, Symposium, Review, etc...) are generated on a different schedule and may not be made available as quickly.

Soon after your final files are uploaded, the early version of your manuscript will be published online unless you opted out of this process. The date of the early version will be your article’s publication date. The final article will be published to the same URL, and all versions of the paper will be accessible to readers.

For Research Articles, you will receive an invoice from PLOS for your publication fee after your manuscript has reached the completed accept phase. If you receive an email requesting payment before acceptance or for any other service, this may be a phishing scheme. Learn how to identify phishing emails and protect your accounts at https://explore.plos.org/phishing.

Thank you again for supporting open-access publishing; we are looking forward to publishing your work in PLOS Neglected Tropical Diseases.

Best regards,

Shaden Kamhawi

co-Editor-in-Chief

PLOS Neglected Tropical Diseases

Paul Brindley

co-Editor-in-Chief

PLOS Neglected Tropical Diseases
